# Proteomic analysis of mouse liver lesions at all three stages of *Echinococcus granulosus* infection

**DOI:** 10.1371/journal.pntd.0012659

**Published:** 2024-12-03

**Authors:** Nan Jiang, Yang Chen, Teng Li, Yeting Sun, Yaxin Su, Ying Wang, Yujuan Shen, Jianping Cao

**Affiliations:** 1 National Key Laboratory of Intelligent Tracking and Forecasting for Infectious Diseases, National Institute of Parasitic Diseases at Chinese Center for Disease Control and Prevention, Chinese Center for Tropical Diseases Research, Shanghai, China; 2 Key Laboratory of Parasite and Vector Biology, National Health Commission of the People’s Republic of China, Shanghai, China; 3 World Health Organization Collaborating Centre for Tropical Diseases, Shanghai, China; 4 The School of Global Health, Chinese Center for Tropical Diseases Research, Shanghai Jiao Tong University School of Medicine, Shanghai, China; 5 State Key Laboratory of Cell Differentiation and Regulation, College of Life Science, Henan Normal University, Xinxiang, China; Universidad de la República Uruguay: Universidad de la Republica Uruguay, URUGUAY

## Abstract

*Echinococcus granulosus*, a zoonotic parasite, can severely damage host health or even lead to host death. In humans, early diagnosis of *E*. *granulosus* infection is difficult because the initial stages of the infection tend to be asymptomatic, this delays treatment and worsens prognosis in most patients. Herein, we present a comprehensive, temporal proteomic atlas of the liver at three stages of *E*. *granulosus* infection and analyze the changes in the proteome of host focal lesions; this atlas may provide an overview of the effects of *E*. *granulosus* in the host, as well as the interactions between them. We identified 3,197 proteins from mice model at 1, 3, and 6 months after *E*. *granulosus* infection; of these proteins, 760 were differentially expressed (520 upregulated; 240 downregulated). Moreover, 228 differentially expressed proteins were screened through cluster analysis and classified into four clusters according to their changing trends. Subsequently, candidate molecules related to cyst invasion, growth, candidate pathways and proteins related to angiogenesis were noted to demonstrate important value in mouse liver. Next, we used western blotting to verify the presence of the aforementioned proteins in mouse liver. In the later stages, *E*. *granulosus* infection was noted to result in significant enrichment of crucial proteins facilitating protoscoleces growth and development and inhibition of amino acid and lipid metabolic enzyme expression in mouse liver; it was also noted to transform host metabolism by weakening oxidative phosphorylation and enhancing glycolysis. In conclusion, we explored the molecular mechanisms underlying the parasitic processes of *E*. *granulosus* through proteomic analysis. Our results provide evidence that may enable the exploration of core regulatory targets for early and effective diagnosis and immunotherapy of *E*. *granulosus* infection, as well as parasite–host interactions involved in cystic echinococcosis development.

## Author summary

Infection with *Echinococcus granulosus*, a life-threatening zoonotic parasite, can remain asymptomatic for a long time; it is generally detectable only in the advanced stages, which delays treatment in most patients. Here, we aimed to determine what *E*.*granulosus* protoscoleces infection does to the host organs and how *E*. *granulosus* evades the host immune system and remains undetectable for a long time, and eventually understand the interactions between *E*. *granulosus* and the hosts in depth. Therefore, we established a mouse model of *E*. *granulosus* infection at the early, middle, and late stages, obtained samples from its liver (the most frequently affected host organ), and performed comparative, quantitative proteomic analyses. Our results indicated the potential changes in the host lesion proteome during the development of *E*. *granulosus* infection. They may aid in developing methods for early and effective diagnosis of cystic echinococcosis and identifying relevant core regulatory targets of parasite–host interactions.

## Introduction

*Echinococcus granulosus sensu lato* is a zoonotic parasite. Its adult worms dwell in the small intestine of definitive hosts, including canids, whereas its larvae, called hydatid cysts, infect the livers and lungs of humans and livestock and cause cystic echinococcosis (CE) [1,2]. Globally, CE has been associated with a substantial financial burden and serious public health concerns [3].

CE is a chronic disease, mainly accompanied by severe intrahepatic lesions. After hatching from eggs within a host, *E*. *granulosus* oncospheres migrate to its liver, where they develop into hydatid cysts. The hydatid cysts have two protective layers that defend against host immunity; inside these layers is a germinal layer that produces protoscoleces. Protoscoleces have the characteristics of bidirectional development, which either produce numerous new hydatid cysts in an intermediate host or develop into adults in a terminal host [4]. Hydatid cysts grow extremely slowly and thus are not perceived by the host immune system until they grow to a sufficiently large size; as such, most hosts present the symptoms 5–20 years after *E*. *granulosus* infection [5,6]. Because of a lack of obvious clinical symptoms during the aforementioned incubation period [7], along with the unavailability of appropriate methodologies for early diagnosis, patients are diagnosed at a later stage of the infection, which often results in serious consequences. CE mainly affects the liver (70%), followed by the lungs (20%) [8]. The interactions and mechanisms underlying the preference of hydatid cysts for parasitizing the liver warrant exploration.

Proteomic technology, in combination with image data analysis, mass spectrometry (MS), and bioinformatic analysis, has been applied in various domains for its superior protein separation capability. In recent years, parasite proteomics has gradually attracted the attention of scholars; for instance, several studies have performed proteomic analysis on the hydatid fluid, hydatid cyst, and protoscoleces of *E*. *granulosus* [9]. Of all assessed *E*. *granulosus* sample types, hydatid fluid is used most frequently because of its antigenicity. A study compared hydatid fluid proteomes from sheep, cattle, and humans and reported similarity among the proteins identified across all three host species; however, the hydatid fluid proteome from sheep demonstrated the highest protein abundance, and only the hydatid fluid proteome from cattle demonstrated the presence of parasite heat shock protein and annexin A13 within cysts that were infertile possibly due to reasons closely related to cellular apoptosis and stress [10]. The EgAgB family proteins, the most common antigens in hydatid cysts, are mainly localized in the germinal layer [11]. It is a potential ligand for monocyte and macrophage receptors that regulate plasma lipoprotein recognition; moreover, it can induce an anti-inflammatory phenotype when recognized by macrophages [12]. A recent study indicated that EgAgB could be used for serological screening of *E*. *granulosus* infection in sheep [13]. Comparative proteomics has also been used to study the proteins in protoscoleces; in total, 550 unique proteins, particularly antioxidant proteins (e.g., glutathione S-transferases and aldo–keto and carbonyl reductases), have been identified in protoscoleces. These data may aid in elucidating molecular mechanisms underlying oxidative stress in *E*. *granulosus* [14]. Although proteomics technology has become a crucial platform for characterizing protein function based on its own predominance, it has not been fully developed for or applied to parasites. In particular, the current knowledge regarding molecular mechanisms underlying parasite–host interactions in both major organs and during disease progression remains limited; thus, obtaining an in-depth understanding of the relevant interaction patterns and identifying the related key molecules along with their potential functions are essential.

In the present study, we used a quantification method based on high-sensitivity liquid chromatography (LC)–tandem MS (MS/MS) proteome, combined with bioinformatics technology, to comprehensively analyze the proteomic differences in the hepatic focal lesions of infected and uninfected mice at different stages of *E*. *granulosus* infection. The accuracy of our proteomic analysis was further verified using mouse livers through western blotting. Our findings facilitated the discovery of crucial candidate molecules in *E*. *granulosus* infection that may be used for diagnosing *E*. *granulosus* infection early, understanding the immune evasion mechanisms of *E*. *granulosus*, identifying effective drug targets in the parasite, and developing vaccines.

## Materials and methods

### Ethics statement

All animal experiments were approved by the Laboratory Animal Welfare & Ethics Committee of the National Institute of Parasitic Diseases, Chinese Center for Disease Control and Prevention (approval no.: IPD-2020-15).

### Animals

Female BALB/c mice (age = 4–6 weeks) were purchased from Jihui Laboratory Animal Care (Shanghai, China) and raised in aseptic conditions with ad libitum food and water supplementation. All animals were first acclimated to the laboratory environment for 1 week; then, they were divided into infection and control groups.

### *E*. *granulosus* protoscolex collection and infected mouse model establishment

We obtained *E*. *granulosus* protoscoleces (G1 genotype) by extracting the hydatid fluid from fertile hydatid cysts in the livers of naturally infected sheep from Xinjiang Uygur Autonomous Region, China. After collection, the protoscoleces were washed five times with sterile 0.9% NaCl containing penicillin (100 U/mL) and streptomycin (100 μg/mL). Protoscoleces with >95% viability, identified using a trypan blue solution, were used to construct our mouse models.

Anesthetized mice were fixed on an anatomic plate, next, their abdominal cavities were opened under aseptic conditions, and a part of their liver lobes was exposed. As an infected group, 100 μL of 0.9% NaCl containing 200 live *E*. *granulosus* protoscoleces were injected onto the liver surfaces of mice (n = 18); in contrast, 100 μL of 0.9% NaCl without any protoscoleces was injected onto the liver surfaces of the uninfected group mice (n = 18). Next, sterile suturing was performed. Finally, the mice from both groups were returned to their cages with ad libitum food and water access.

### Sample preparation and collection

At three timepoints (1, 3, and 6 months) after model establishment, three mice each from the infected and uninfected groups were selected randomly; then, their liver tissue with lesions was collected through dissection after euthanasia. Finally, the tissue was snap-frozen in liquid nitrogen for a few minutes and then transferred to −80°C for storage until further experimentation.

### Protein extraction and trypsin digestion

The liver tissue from each group was separately ground into a powder in liquid nitrogen and transferred to centrifuge tubes. Next, we added four volumes of lysis buffer cocktail containing 8 M urea and 1% protease inhibitor. Thereafter, the tubes were sonicated three times on ice in an ultrasonic processor (Scientz, Zhejiang, China), followed by centrifugation at 12,000 *× g* at 4°C for 10 min. Next, the supernatant was aspirated and used for determining protein concentration by using a BCA kit (Beyotime, Jiangsu, China). Finally, a protein supernatant of a known concentration was completely digested with sequencing-grade modified trypsin (*V5117*; Promega) for further analysis.

For trypsin digestion, the protein solution was precipitated at −20° for 2 h by adding 1 volume of precooled acetone, vortex mixing, and then adding four volumes of precooled acetone. After centrifugation at 4,500*× g* at 4°C for 5 min, the supernatant was discarded, and the precipitate was washed twice with precooled acetone, followed by the addition of 200 mM tetraethylammonium bromide. Finally, trypsin was added at 1:50 trypsin-to-protein mass ratio, followed by incubation at 37°C overnight. Then, this mixture was reduced using 5 mM dithiothreitol at 56°C for 30 min and alkylated with 11 mM iodoacetamide at room temperature for 15 min in the dark. Next, we adjusted the pH of the peptide solution to 2–3 with 10% trifluoroacetic acid (TFA) and centrifuged at 12,000 × *g* at room temperature for 10 min. The supernatant was transferred to a new EP tube, and the solid phase extraction column was activated with methanol and balanced with 0.1% TFA. Finaly, the acidified peptide solution was loaded onto the solid phase extraction column, desalted with 0.1% TFA, and eluted with 80% acetonitrile.

### LC-MS/MS analysis

The amount of protein prepared for each sample was 2,170 μg, and the amount of peptides used for each sample was 1,740 μg. The tryptic peptides were loaded onto a reversed-phase analytical column (length = 25 cm, inner diameter = 75 μm). Peptides were separated at a constant flow rate of 500 nL/min on an EASY-nLC 1200 ultra-performance LC system (Thermo Fisher Scientific) using three gradients: 4%–23% over 60 min, 23%–35% in 22 min, 35%–80% in 4 min, and 80% in 4 min. The separated peptides were analyzed on a Q Exactive HF-X system (Thermo Fisher Scientific) with a nanoelectrospray ion source (parameters: electrospray voltage, 2.1 kV; scan resolution, 120,000; scan range: 400–1,500 *m/z*). Up to 10 most abundant precursors were then selected for further MS/MS analyses. The fragments were surveyed in an Orbitrap at a resolution of 15,000 with 100 m/z of the first mass and the automatic gain control target set at 5E4.

### Database search

The MS data (three repeats), which concatenated with the reverse decoy database, were analyzed using the search engine MaxQuant (v.1.6.15.0); a search was performed against Mus_musculus_10090_SP_20210721.fasta (*Mus musculus* database downloaded from UniProt in July 2021). Trypsin/P was specified as the cleavage enzyme, with allowance for up to two missing cleavages. The minimum length of peptides after trypsinization was set at seven amino acid residues. The mass tolerance was set separately at 20 ppm for precursor ions in the first search and at 4.5 ppm for fragment ions in the main search. The mass error tolerance was set at 0.02 Da for secondary fragment ions. The false discovery rate of proteins, peptides, and peptide-spectrum matches was adjusted to <1%, with the minimum number of unique peptides set at ≥1.

The differentially expressed proteins (DEPs) were identified using the two-tailed Fisher exact test, with the following criteria as the threshold for significant change: *P* < 0.05 and fold change (FC) > 1.5 or FC < 0.6667.

### Bioinformatic analysis

Gene Ontology (GO) annotation proteome results were derived from the database UniProt-GOA (**http://www.ebi.ac.uk/GOA/**). The protein sequences were searched to determine their potential function by using the domain database InterPro (**http://www.ebi.ac.uk/interpro/**). Biological process pathways or annotation were assessed using online service tools for describing proteins in the Kyoto Encyclopedia of Genes and Genomes (KEGG) database (**http://www.genome.jp/kaas-bin/kaas_main, http://www.kegg.jp/kegg/mapper.html**). The pathways related to the DEPs were classified into hierarchical categories based on the KEGG database. All these results were clustered in Genesis and visualized using the R package pheatmap **(https://cran.rproject.org/web/packages/cluster/).**

We then predicted the subcellular localizations of the identified proteins (http://www.genscript.com/psort/wolf_psort.html). To analyze protein–protein interactions (PPIs), we searched and aligned all the identified proteins’ accession numbers or sequences with the STRING database (version 11). The selection was limited to the search dataset, excluding the external data; only PPIs with confidence score ≥ 0.7 (i.e., high confidence) were selected, and the resulting networks were visualized using Cytoscape. The Mfuzz method was used for cluster analysis, where the number of clusters was set at 4 and the degree of clustering ambiguity at 2. The software package heatmap gplots was used to visualize the clustering results. Finally, we used a protein gene set enrichment analysis (GSEA) website (https://www.gsea-msigdb.org/gsea/index.jsp) for assessing concerned gene distribution trends and their influence on phenotypic changes.

### Histopathology

After excision, liver tissue was immediately fixed in 4% paraformaldehyde, treated in an ethanol gradient, and finally embedded in paraffin using conventional methods. Paraffin-embedded tissues were sectioned into 4-μm-thick slices and stained with the hematoxylin and eosin dye (H&E; Wuhan Servicebio Technology, China), according to the manufacturer’s instructions. Images of these sections were recorded at 20× magnification; they are presented with scale bars of 50 μm.

### Western blotting

Proteins were extracted from liver tissue from the infected and uninfected groups, as described above. Next, 20 μg of protein was separated per lane through 12% sodium dodecyl sulfate polyacrylamide gel electrophoresis (P0012A; Beyotime, China) and transferred onto a polyvinylidene fluoride (PVDF) membrane (0.22 μm; Bio-Rad, USA). Then, the membrane was blocked at room temperature for 20 min by using a protein-free fast-blocking solution (PS108; Shanghai Epizyme Biomedical Technology, Shanghai, China). Next, tris-buffered saline containing 0.5‰ Tween-20 (TBST) was used to wash all PVDF membranes five times for 10 min each. These membranes were then incubated with primary antibodies at 4°C overnight with gentle shaking. Here, we used a primary rabbit monoclonal antibody (4970S, Cell Signaling Technology, CST, USA), as well as primary antibodies against β-Actin (13E5), 6-phosphofructokinase (muscle type) (PFKM; 55028-1-AP; Proteintech, USA), fructose-bisphosphate aldolase A (ALDOA; 11217-1-AP; Proteintech), collagen type VI alpha 1 (COL6A1; 17023-1-AP; Proteintech), extracellular regulated protein kinases 1/2 (ERK1/2; 11257-1-AP; Proteintech), lactate dehydrogenase (LDH) B (LDHB; 14824-1-AP; Proteintech). Next, membranes were washed five times with TBST and then incubated with corresponding secondary antibodies at room temperature for 1.5 h. The immune complexes were then visualized using an enhanced chemiluminescence kit (SQ201; Epizyme). The densities of the immunoreactive protein bands were determined using Image J. The gray value of the target protein is divided by the gray value of the internal reference (β-actin) to correct errors. The final values represent the relative expression of the target proteins.

### Statistical analysis

All statistical analyses were performed using GraphPad Prism (version 8.0; GraphPad, La Jolla, CA, USA). Data are presented as means ± standard deviations (SDs). A *P* value of <0.05 was considered to indicate statistical significance.

## Results

### Histopathological analysis of livers from mice infected with *E*. *granulosus*

**Fig 1:**
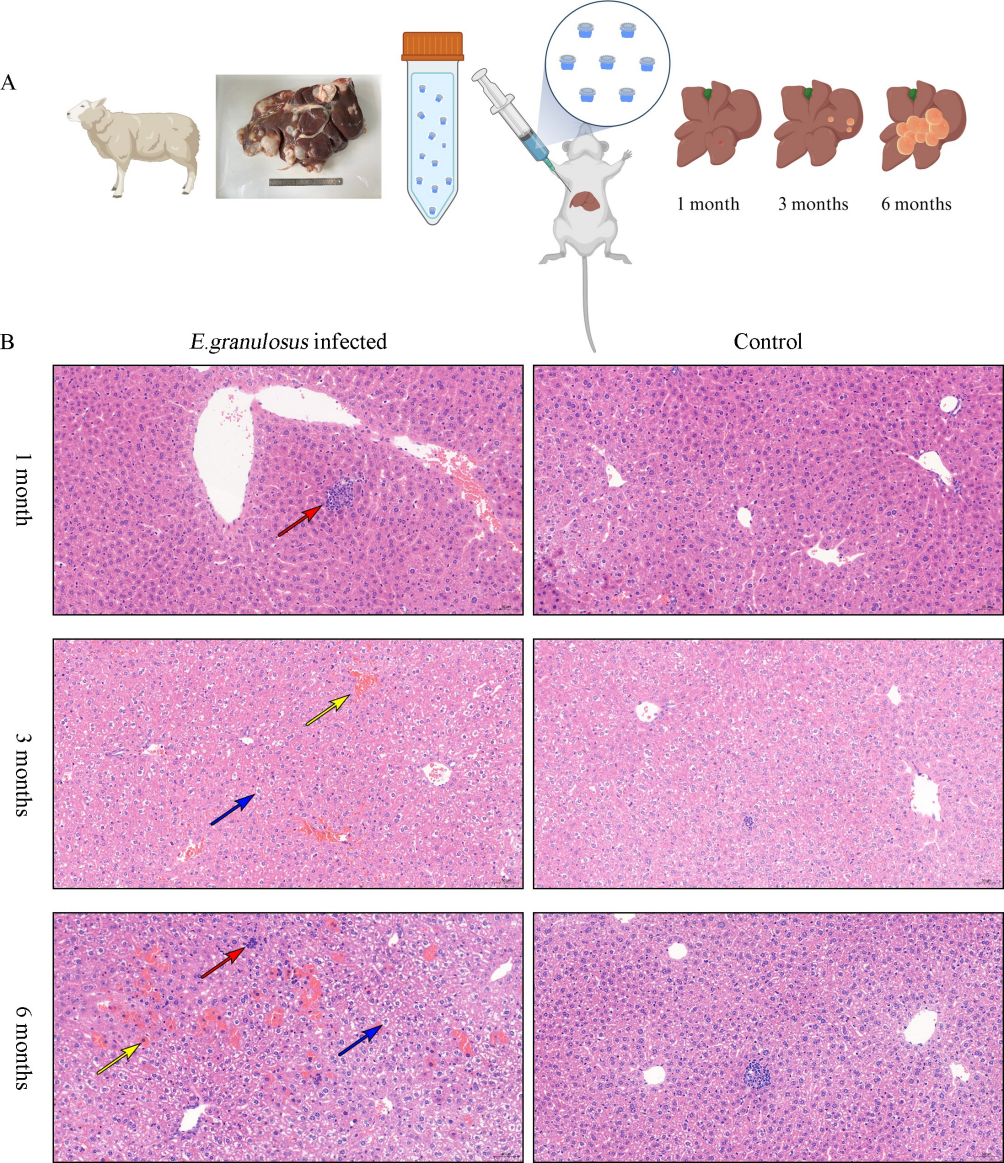
Histopathological analysis of liver in mice infected with *E*. *granulosus*. (A) Sample preparation process. BALB/c mice were intrahepatically inoculated with a suspension of *E*. *granulosus* protoscoleces obtained from the liver of naturally infected sheep in an endemic area. Liver tissue with lesions was collected at specific postinfection timepoints (including liver tissues from the uninfected group injected with 0.9% NaCl; not shown) for H&E staining. The materials were created in BioRender.com. (B) H&E staining of *E*. *granulosus*-infected and uninfected (control) mouse livers at different infection stages. Focal infiltration of lymphocytes (red arrow) was occasionally noted in the hepatic lobules 1 month after infection. Eosinophilic granules (blue arrow) were noted in the cytoplasm, and a small amount of venous congestion (yellow arrow) was observed 3 months after infection. Vacuolization of cytoplasm (blue arrow), few extramedullary hematopoietic foci (red arrow), and red blood cells (yellow arrow) appeared in the local areas 6 months after infection.

After establishing the animal model (Fig 1A), we assessed the lesions on the livers of mice at different infection times (S1 Fig) and found that the liver lesions were not evident in the early stage (1 month). In the middle stage (3 months), white, translucent hydatid cysts (diameter ≈ 3 mm) with clear boundaries appeared to surround liver tissues. In the late stage (6 months), multiple transparent cysts of different sizes protruding from the liver surface were observed; they were difficult to peel off. Paraffin-embedded liver tissue sections demonstrated focal infiltration of lymphocytes (red arrow) occasionally in the hepatic lobules in the early stage of infection. In the middle stage of infection, moderate granular degeneration of hepatocytes appeared widely in the liver tissue; eosinophilic granules (blue arrow) were noted in the cytoplasm, along with a small amount of venous congestion (yellow arrow). In the late stage of infection, we observed increased balloon-like hepatocyte degeneration, cytoplasm vacuolization (blue arrow), and few extramedullary hematopoietic foci (red arrow) in the hepatic lobules, along with red blood cells (yellow arrow) in the cytoplasm of many hepatocytes in the local area (Fig 1B).

### Overview of proteomic identification in mouse livers from infected and uninfected groups

**Fig 2:**
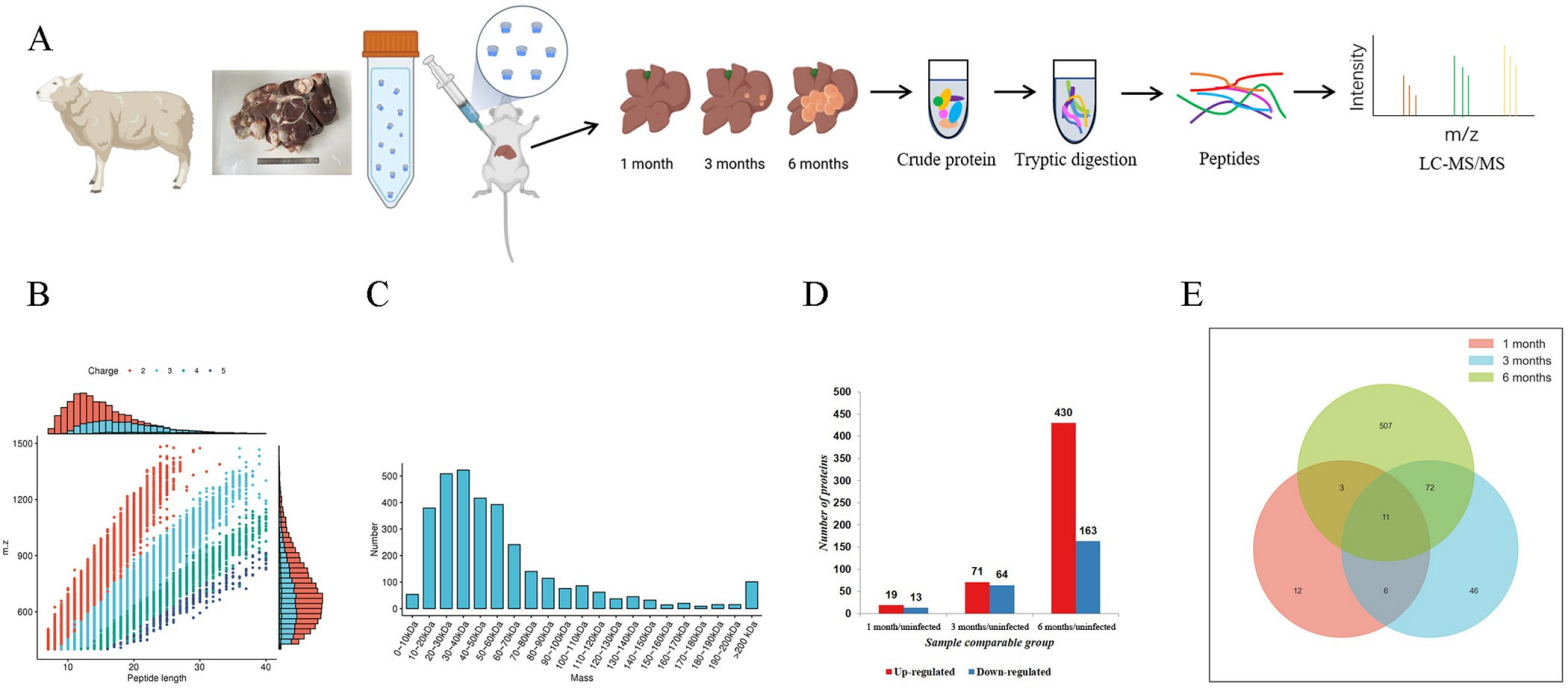
Overview of Proteomic Identification in Infected and Uninfected Group Liver Tissue. (A) The mouse model was constructed as mentioned above. Liver tissue with lesions was collected (including uninfected group) at 1, 3, and 6 months after infection for LC-MS/MS analysis. (B) Distribution of peptide length among DEPs in liver tissue from mice infected with *E*. *granulosus* at different infection stages. (C) Molecular weights of DEPs at different infection stages. (D) Histogram of DEPs at different infection stages. (E) Venn diagram of DEPs at different infection stages. Created with Biorender.com.

We examined the liver samples of the mice in the early (1 month), middle (3 months), and late (6 months) stages of *E*. *granulosus* infection (Fig 2A) and identified a total of 3,197 proteins, of which 2,390 were quantifiable (S2A Fig); moreover, most of the peptides were 7–20 amino acids long (Fig 2B). The molecular weights of the identified proteins, ranging from 6.6 to 3,906.4 kDa, were distributed uniformly at different stages (Fig 2C). By comparing the MS data of the infected and uninfected groups, we identified 760 DEPs throughout the *E*. *granulosus* infection process (FC > 1.5 or FC < 0.6667). Of these DEPs, 520 were upregulated (19, 71, and 430 in the early, middle, and late stages, respectively), and 240 were downregulated (13, 64, and 163 in the early, middle, and late stages, respectively; Fig 2D). Moreover, 11 DEPs were expressed persistently in all three stages of infection (Fig 2E; thedetails of the Venn diagram are shown in S1 Table), with 6 and 3 being persistently upregulated and downregulated, respectively. All the detected proteins conformed to the general rules based on enzymatic hydrolysis and MS fragmentation, and the results met the quality control requirements. In addition, three statistical methods, such as Pearson correlation coefficient (PCC) analysis, principal component analysis (PCA), and relative standard deviation (RSD), were used to evaluate sample repeatability (S2B–S2D Fig).

### Bioinformatic prediction of hepatic proteomic data in host

#### Subcellular localization of DEPs

In the early stage of *E*. *granulosus* infection, the upregulated DEPs were mainly distributed in the extracellular matrix (ECM), cytoplasm, and nucleus (S3A Fig), whereas the downregulated DEPs were mainly located in the plasma membrane (S3B Fig). However, the number of DEPs localized in the cytoplasm was the largest in the middle (S3D Fig) and late (S3E Fig) infection stages, followed by the extracellular in the middle infection stage or nucleus in the late infection stage. The proportion of DEPs localized in mitochondria increased from 3.12% in the early infection stage to 14.7% in the late infection stage (S3C–S3E Fig), suggesting the importance of mitochondria as a location for the regulation of host–parasite interactions. In addition, DEPs located in the cytoskeleton were downregulated in the early stage of infection but upregulated in the middle and late stages (S2 Table). In contrast, no DEP was located on the peroxisome in the early stage, but some DEPs appeared in the middle and late stages (S2 Table).

#### GO and KEGG pathway enrichment analysis of DEPs

**Fig 3:**
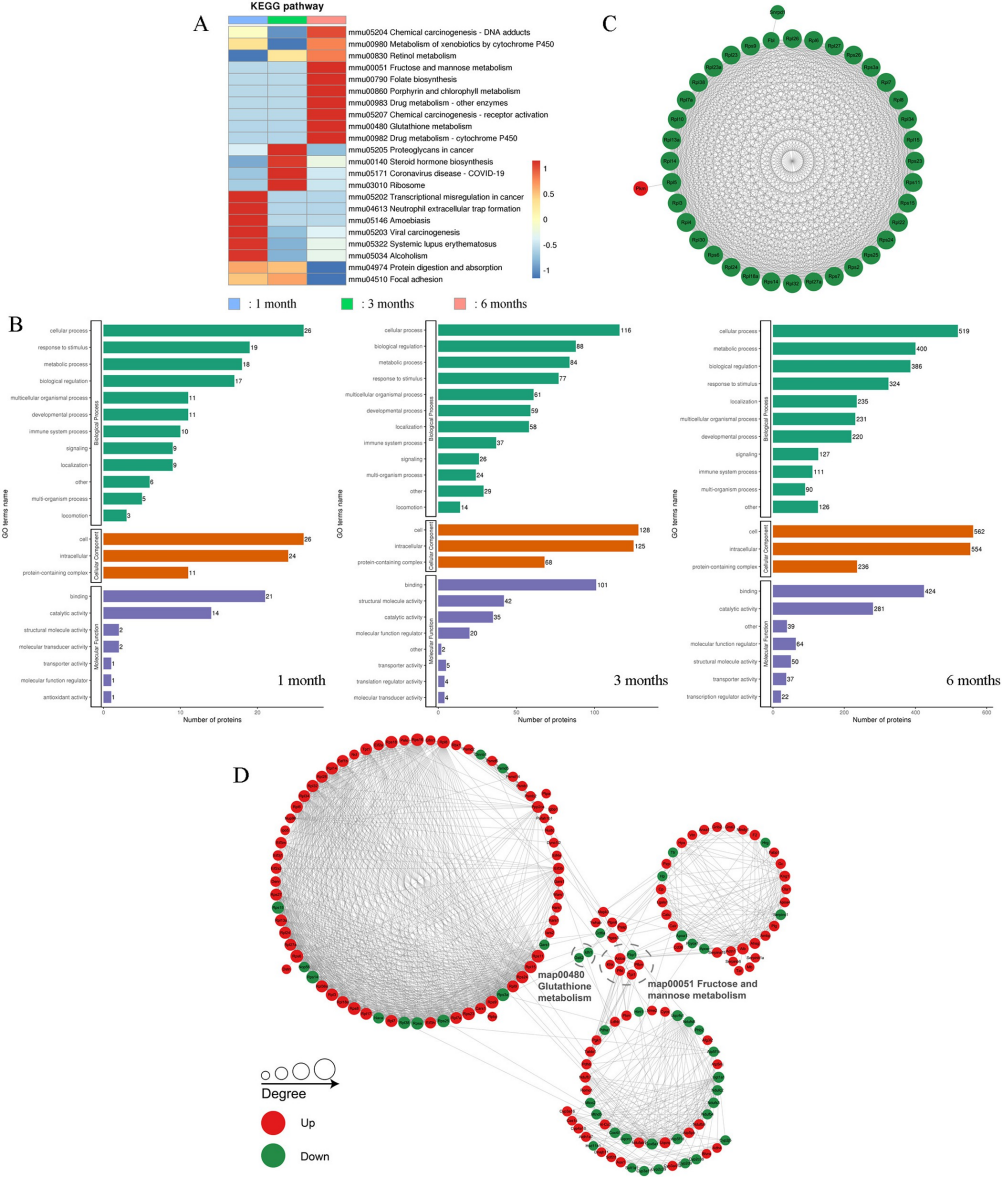
GO and KEGG Pathway Enrichment and PPI Network Analysis After *E*. *granulosus* Infection. (A) Heatmap of DEPs enriched in KEGG pathways. The *y*-axis represents the groups at 1, 3, and 6 months after infection, whereas the *x*-axis represents the description of the KEGG pathways at 1, 3, and 6 months after infection; red and blue represent strong and weak enrichment, respectively. (B) GO classification of DEPs at 1, 3, and 6 months after infection in the Cellular Component, Molecular Function, and Biological Process categories. (C) PPI subnetwork, mainly composed of ribosomal protein families (mainly downregulated DEPs), at 3 months after infection. (D) PPI network of DEPs at 6 months after infection. Circles represent DEPs; the colors represent specific differences in proteins: green = downregulation and red = upregulation.

To understand the key role of the DEPs in the *E*. *granulosus* infection process, we specifically assessed the functions of the DEPs in detail, thus elucidating functional molecules and signaling pathways essential for *E*. *granulosus* infection (Fig 3A). The results suggested that in mouse model, in terms of biological processes, the DEPs were closely related to cellular processes, responses to stimuli, metabolic processes, and biological regulation (Fig 3B). In the functional enrichment analysis, we found that the DEPs had certain molecular functions, such as binding, catalysis, molecular regulation, and transport, in all three stages of infection. The number of proteins increased in a time-dependent manner, indicating that various physiological activities of the host become altered after *E*. *granulosus* infection. In addition, the host immune system was noted to be activated by the parasite. Furthermore, the GO enrichment results suggested that *E*. *granulosus* infection leads to a series of changes in the biological process of the host. The upregulated DEPs were noted to be involved in many aspects: protein complex oligomerization, and leukocyte and lymphocyte activation regulation in the early infection stage; cell migration and motility regulation as well as lipid biosynthetic processes in the middle stage, and the negative regulation of protein metabolic process and neurogenesis in the late stage. By contrast, the downregulated DEPs were mainly associated with synthesis (cellular macromolecule and amide biosynthetic processes) or metabolism (cellular protein and peptide metabolic processes; S4A Fig) in the middle stage with various metabolic processes (carboxylic acid and cellular amino acid metabolic processes; S4B Fig) in the late stage. Therefore, in the late stage (with the largest number of enriched pathways), eight signaling pathways related to metabolism were detected (S4C Fig); moreover, the related DEPs, which were all downregulated, included metabolism of xenobiotics by cytochrome P450 and glutathione and retinol metabolism. These results indicated that *E*. *granulosus* infection induces changes in the host’s hepatic metabolism; these findings may facilitate the discovery of prognostic indicators for *E*. *granulosus* infection.

### PPI network analysis of DEPs after *E*. *granulosus* infection

We screened out the top 50 most closely interacting proteins and mapped their PPI network to intuitively understand diverse, close relationships between the proteins. We noted that the PPIs were not evident in the early infection stage; only four protein clusters appeared in the middle infection stage, with one of the subnetworks being mainly related to the ribosomal protein (S and L subunits) families and composed of 37 downregulated DEPs (Fig 3C). Whether this ribosomal protein dysregulation can be used as an indicator in the middle infection stage warrants further exploration. Stronger PPI networks emerged in the late infection stage (Fig 3D). Several core proteins with potential roles in *E*. *granulosus* infection models were also discerned; for instance, we noted increases in the expression of ALDOA, albumin (ALB), and serine/threonine-protein phosphatase 2A catalytic subunit alpha and decreases in the expression of UDP-glucuronosyltransferase 1A1 (UGT1A1), cytochrome c oxidase subunit 6A1 (COX6A1).

### Clustering analysis of DEPs and GSEA during three *E*. *granulosus* infection stages

**Fig 4:**
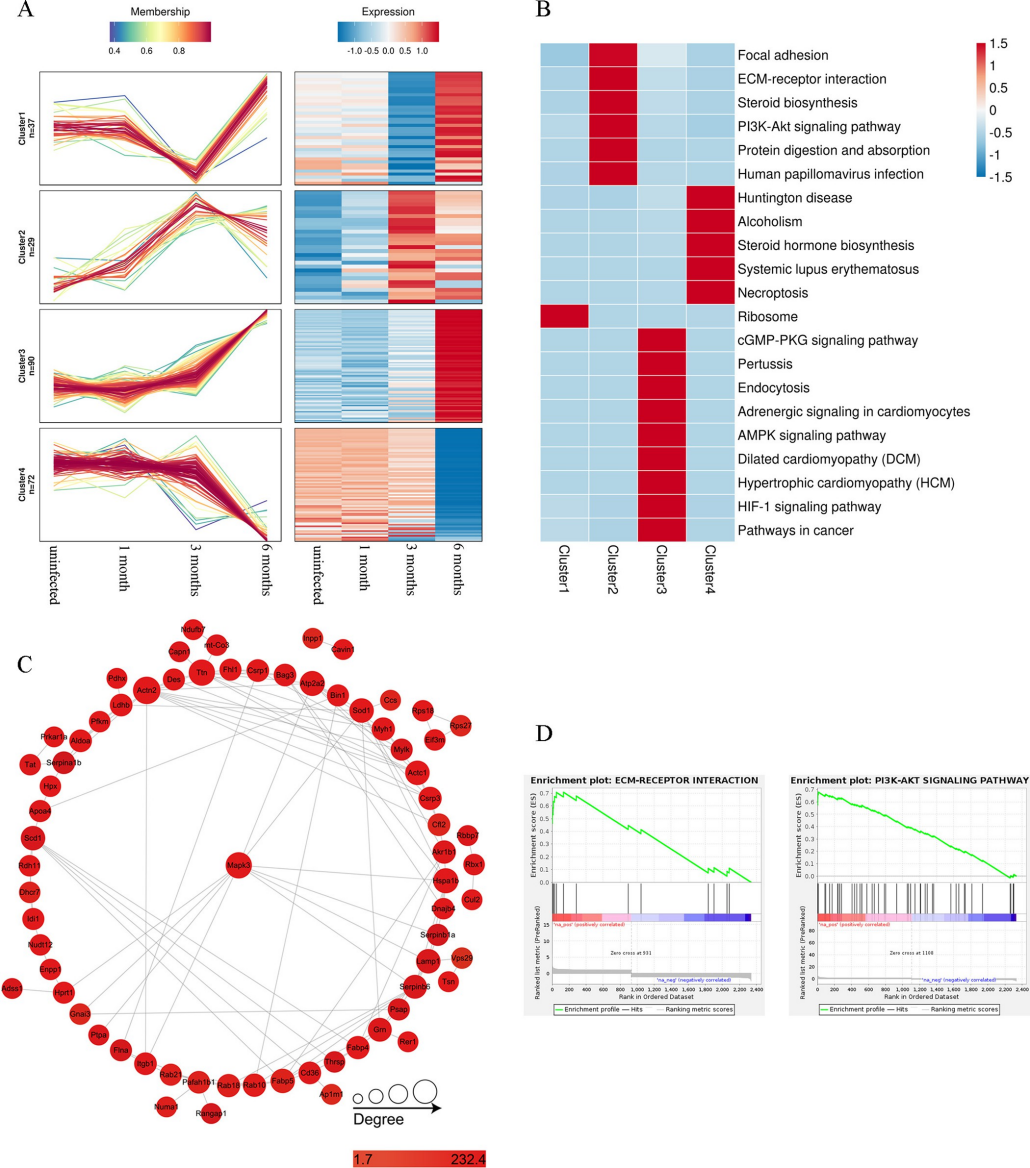
Bioinformatic Analysis of DEPs in Mouse Liver After *E*. *granulosus* Infection. (A) Cluster analysis for significant changes in DEPs in consecutive samples at three *E*. *granulosus* infection stages. The DEPs were divided into four clusters. (Left) A line chart of protein expression; the *x*-axis represents the sample, the *y*-axis represents the relative protein expression levels, and each fold line represents a protein. (Right) Expression heatmap corresponding to the line chart. (B) Clustering heatmap of enriched KEGG pathways. The *x*-axis direction represents four clusters, whereas the *y*-axis denotes the KEGG pathways in which DEPs in different clusters are enriched; red and blue represent strong and weak enrichment, respectively. (C) PPI network analysis for DEPs in Cluster 3. (D) Enrichment plot of two common pathways involving upregulated DEPs. (Left) A representative diagram of the ECM–receptor interaction pathway in the early infection stage. (Right) A representative diagram of the PI3K-Akt pathway in the middle infection stage.

To identify proteins associated with *E*. *granulosus* infection, pathogenicity, or host anti-*E*. *granulosus* response at the various infection stages, we used the Mfuzz method for clustering analysis for changes in protein abundance at different timepoints after infection. Of all 3,197 proteins, 228 were selected and categorized into four clusters; proteins in each cluster exhibited similar expression trends during the three *E*. *granulosus* infection stages (Fig 4A). Expression of Cluster 1 significantly decreased in the middle stage but increased in the late stage, that of Cluster 2 increased in the middle stage, that of Cluster 3 significantly increased in the late stage, and that of Cluster 4 decreased in the late stage. We then performed KEGG pathway enrichment analysis for each cluster (Fig 4B); the results indicated that Cluster 1 primarily comprised ribosomal proteins, consistent with our PPI results (Fig 3C); Cluster 2 included proteins involved in various pathways, such as focal adhesion, ECM–receptor interactions, and Pl3K-Akt signaling pathway; Cluster 3 comprised proteins involved in the cGMP-PKG, AMPK, and hypoxia-inducible factor 1 (HIF1) pathways; and Cluster 4 included proteins involved in steroid hormone biosynthesis and necroptosis. Our results, based on the animal model and proteomics data, indicated that the effects of *E*. *granulosus* parasitism in the host are mild in the early stages, but they worsen with disease progression. Thus, specific genes in Cluster 3 and their relationships with KEGG functional enrichment pathways are further displayed in our circle plot (S4D Fig), along with the analysis of their PPI network (Fig 4C).

Next, GSEA was performed to intuitively understand the expression of specific proteins in each pathway during the entire infection process (S5 Fig). Numerous signaling pathways were found to be affected by *E*. *granulosus* infection, with upregulated and downregulated DEPs involved in different enriched KEGG pathways. Notably, during the infection process, the upregulated DEPs primarily participated in ECM–receptor interactions and the PI3K-Akt pathway (Fig 4D), and the amounts of DEPs enriched in both pathways were the highest at the late infection stage. The significant changes in ECM–receptor interaction pathways mainly included collagen and vitronectin, whereas those in the PI3K-Akt pathway included extracellular regulatory protein kinase (ERK)—also known as mitogen-activated protein kinase (MAPK).

**Fig 5:**
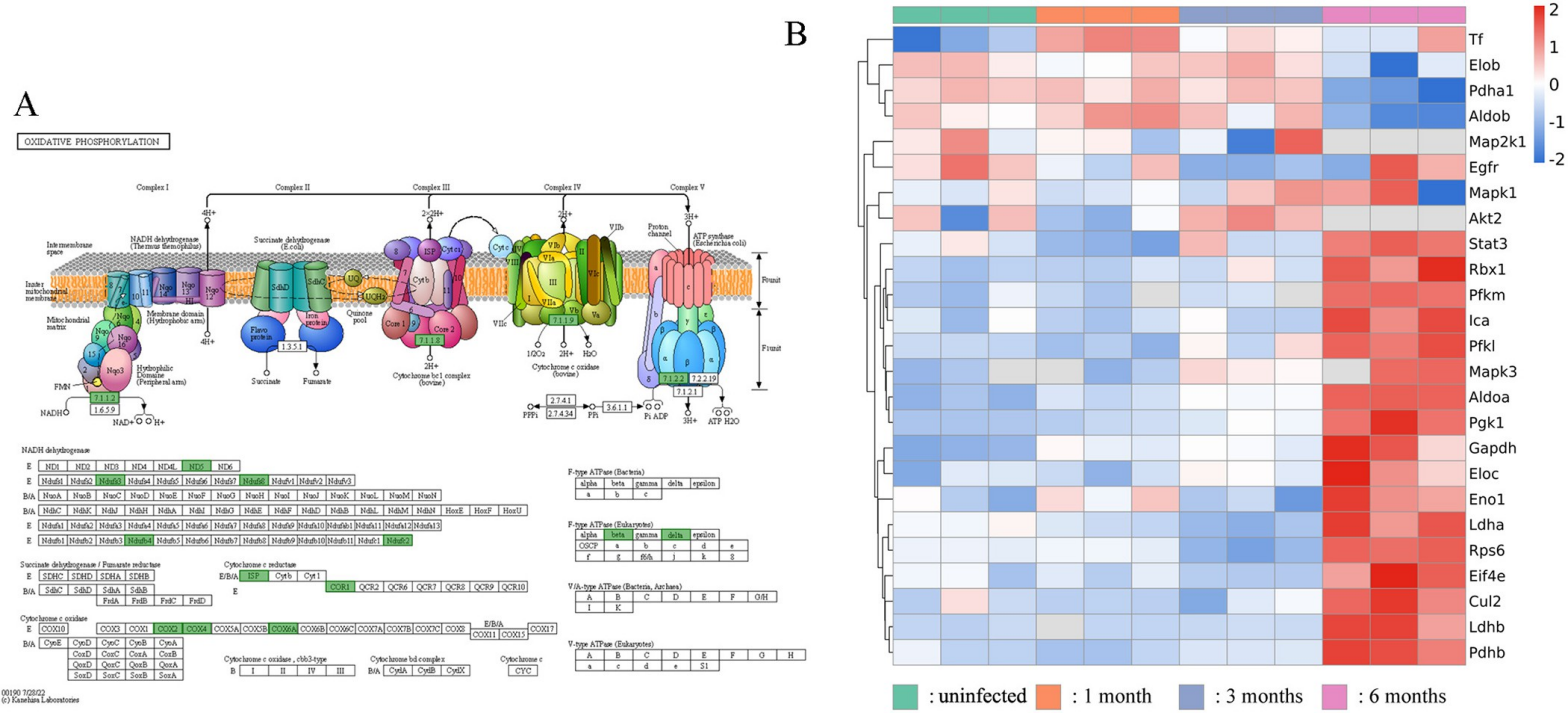
*E*. *granulosus*-Induced Changes in Mouse Metabolism. (A) *E*. *granulosus* affecting oxidative phosphorylation process in the host (a representative diagram at 6 months), with multiple related proteins being downregulated after infection (green). (B) Heatmap of expression of DEPs belonging to the HIF1 pathway in each sample. A significant increase in expression of multiple key proteins was noted 6 months after infection.

At the late *E*. *granulosus* infection stage, significant changes were noted in host reactive oxygen species and oxidative phosphorylation (Fig 5A), with up to 29 related proteins significantly downregulating after infection. Moreover, a notable simultaneous increase was noted in the expression of several key proteins related to anaerobic metabolism from the HIF1 pathway (Fig 5B), including pyruvate dehydrogenaseβ (PDHb), LDH, phosphofructokinase, ALDOA, and phosphoglycerate kinase 1. Thus, in the parasitic microenvironment of *E*. *granulosus*, oxidative phosphorylation may diminish, shifting toward enhanced glycolysis.

### Proteomic data validation

**Fig 6:**
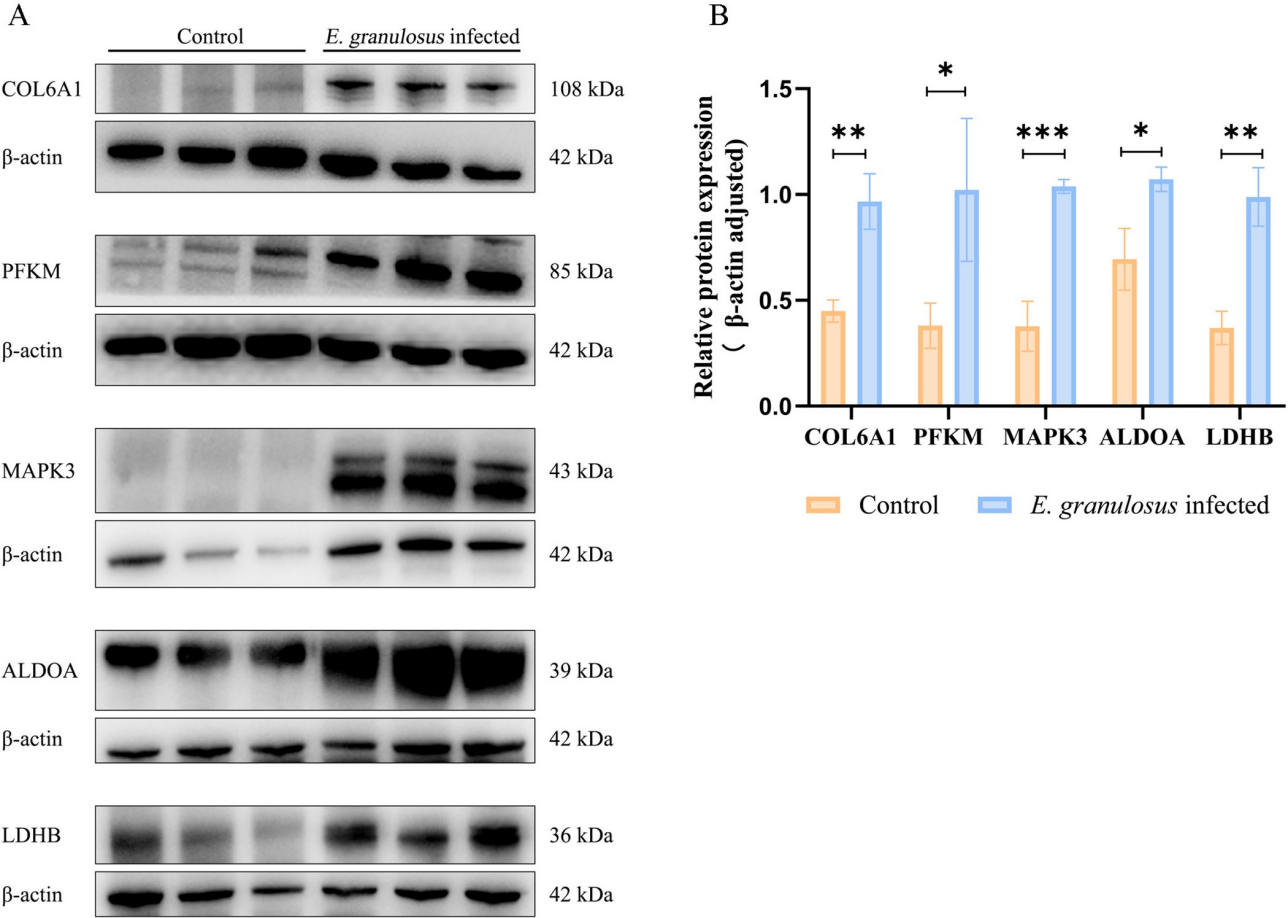
DEP Verification in Infected and Uninfected Groups. (A) Western blots for confirming identified proteins’ expression levels with biological significance in the infected group 6 months after infection (n = 3). (B) Histogram of western blotting results. *P < 0.05, **P < 0.01, ***P < 0.001.

Considering the infection characteristics of our *E*. *granulosus* infection mouse model, we assessed proteins related to its infection, development, and pathogenicity by selecting a few top DEPs for validation through western blotting in both the infected and uninfected groups at 6 months after infection. Our western blotting results demonstrated that COL6A1, PFKM, MAPK3, ALDOA, and LDHB expression corroborated those of our proteomic analysis (Fig 6). The partial proteomic data of validation proteins are listed in S3 Table.

## Discussion

*E*. *granulosus* eggs invade the host to develop into hydatid cysts, which gradually grow and affect the function of surrounding tissues and cells or mechanically oppress adjacent organs. Finally, they induce clinical symptoms such as toxicity, allergy, and secondary infections [15]. Hydatid cysts can be parasitic in many host organs, particularly the liver [2]. Therefore, in this study, we selected the liver of mice as the target organ. Our results demonstrated that host DEP levels changed at three stages of *E*. *granulosus* infection, as described previously. Moreover, these DEPs were noted to demonstrate numerous functions, which indicated the parasite leads to changes in diverse biological processes in the host organs. The DEP number was proportional to the *E*. *granulosus* infection duration, increasing in a time-dependent manner (32, 135, and 593 in the early, middle, and late infection stages, respectively). Thus, the effects of relevant changes on the host are mild in the early *E*. *granulosus* infection stage, and they worsen with the further development of the disease.

This effect was also reflected in the PPI network analysis, the interaction network with strong interaction does not appear until the late stage of infection. We also discerned several core proteins with potential roles; or instance, we noted increases in ALDOA, MAPK3 and ALB expression and decreases in UGT1A1 and COX6A1 expression. ALDOA is a pivotal enzyme in the glycolytic pathway [16], and its knockdown can inhibit tumor growth and migration under hypoxia [17]. In our mouse model, ALDOA was upregulated in the late infection stage and appeared at the PPI core. Thus, ALDOA may be an essential driver of *E*. *granulosus* growth in the late-stage parasitic microenvironment. As such, ALDOA inhibitors, which can limit *E*. *granulosus* development, may be candidates for active antiparasitic drugs. Similarly, MAPK3 was also identified as a core protein in the group of proteins (Cluster 3) with increased expression in the late stage. The MAPK pathway becomes activated in response to various extracellular regulatory signals. However, MAPK3, located downstream of the MAPK pathway is abnormally expressed in many diseases [18]. Selective MAPK3 inhibition is considered a potential therapeutic strategy for these diseases [19]. Therefore, targeting MAPK3 may facilitate the discovery of newer small-molecule inhibitors as candidate drugs for parasitic infection treatment. In the current study, we noted that UGT1A1, ALB, and COX6A1 in the late-infection-stage PPI are associated with liver homeostasis. UGT1A1, the only enzyme that conjugates with bilirubin [20], is significantly downregulated in malignant hepatocellular carcinoma [21]; therefore, it can be used to predict liver cancer risk [22]. Moreover, UGT1A1 was noted to be downregulated in the late infection stage, suggesting that bilirubin accumulation occurred in the mouse liver, resulting in other adverse reactions or hepatotoxicity. ALB can maintain the osmotic pressure–fluid exchange balance [23]; in addition, its serum expression can increase with liver disease severity [24]. COX6A1-deficient mice are highly likely to develop liver dysfunction [25]. As such, we speculate that at the late *E*. *granulosus* infection stage, liver dysfunction results in abnormal changes in UGT1A1, ALB, and COX6A1 expression in mice to some extent. Thus, reversing the expression levels of these proteins (essential for physiological balance in the liver) may promote the restoration of liver function. In summary, ALDOA, MAPK3, UGT1A1, ALB, and COX6A1 may aid in effectively distinguishing *E*. *granulosus* infection stages in animal models. The specific functions and underlying mechanisms of these proteins may represent the most intuitive feedback to *E*. *granulosus* invasion in hosts.

We collected liver tissue samples from our mouse model at three timepoints; the results demonstrated that a few proteins were persistently expressed at all three timepoints, with consistent expression changes (i.e., upregulation or downregulation). Of these proteins, three immunoglobulins [namely immunoglobulin γ 1 heavy chain constant region (IGHG1), immunoglobulin kappa C (IGKC), and IGHM] demonstrated a persistent increase in expression with an increase in infection duration; the upregulation of all three proteins is likely related to the secretion of specific antibodies against *E*. *granulosus* in the liver. The related immunoglobulin genes can promote the development of various diseases [26], and are also associated with the prognoses [27,28] or recurrence [29] of some malignancies. In the current model, the aforementioned immunoglobulins were detected at the early infection stage, and their expression continued to increase until the late stage, indicating the functioning of the host immune system; however, the specific mechanisms underlying *E*. *granulosus* infection remain unknown. We are currently validating these mechanisms in vitro in cell lines so as to confirm their potential for prognosis and targeted therapy of *E*. *granulosus* infection.

IGHG1 has been reported to induce epithelial–mesenchymal transition (EMT) [30, 31]. In the current model, galectin 1 (LGALS1) was noted to be upregulated and associated with EMT [32]. EMT was initially considered to mainly exist in the embryonic development process; nevertheless, it is also involved in various biological processes, such as wound healing, tissue fibrosis, and cancer metastasis and invasion [33]. It can reduce adhesion in cells, causing them to lose their polarity and gain an athletic ability. Mohammed *et al*. proposed that *E*. *granulosus* cyst fluid enhances EMT in human lung epithelial cells [34]. In the current study, the EMT marker vimentin was detected in both the middle and late infection stages, and its expression levels were significantly higher in the infected group than in the uninfected group, indicating that *E*. *granulosus* can induce EMT in host livers. This result is consistent with that reported by Mohammed *et al*. EMT is a core mechanism improving cancer cell motility and invasiveness, induced by collagen [35,36]. Thus, we hypothesize that IGHG1 and LGALS1, detected early in *E*. *granulosus* infection, induce EMT initiation in the host liver cells; this results in vimentin expression in the middle infection stage, followed by persistent increases in expression in the late infection stage. EMT may thus be a crucial mechanism underlying *E*. *granulosus* motility and growth promotion.

*COL6A2*, a typical gene involved in collagen synthesis, can promote fibrosis. As a scaffold for the tumor microenvironment [37], COL6 not only regulates ECM remodeling to promote tumor cell infiltration, migration, and invasion [38], but also builds a fibrous barrier in the ECM to prevent cancer cell proliferation further [39]. In a host, cancer cell growth and development mechanisms are similar to those for hydatid cysts; in both cases, an imbalance occurs between the immune escape and proliferation of invaders and the immune system of the host. As mentioned earlier, one of the characteristics of CE is the development of slow-growing hydatid cysts in the liver or lungs of the host. The survival and growth of these cysts depend on the host–parasite interaction balance and fibrotic substances around the hydatid cyst; even though these factors limit the parasite’s development, they facilitate cyst wall formation that protects the parasite. COL6A2, localized in the ECM, was detected at all three *E*. *granulosus* infection stages; COL6A1 expression increased significantly in the middle and late infection stages. The involvement of COL6A1 expression in ECM development around the lesion, as well as its immunosuppressive effect [40], may explain the reason that clinical symptoms do not appear in patients with CE until several years after infection.

Our GSEA data demonstrated that the upregulated DEPs were concentrated in the ECM–receptor interaction and PI3K-Akt pathways. As mentioned previously, the ECM is critical in various diseases, particularly cancer—where its interaction with receptors can promote and mediate cancer cell proliferation and progression. We noted a significant increase in expression of vitronectin involved in the ECM–receptor interaction pathway at the late *E*. *granulosus* infection stage, promoting endothelial cell adhesion, migration, and proliferation, thereby mediating wound repair and angiogenesis [41]. The PI3K-Akt pathway is activated during cancer cell growth, metastasis, and invasion; thus, it is involved in tumor angiogenesis regulation [42]. In a previous study, we preliminarily confirmed the occurrence of angiogenesis during *E*. *granulosus* infection [43]; here, the exploration of vitronectin and the PI3K-Akt pathway provided the basis for further elucidating its specific mechanism. In brief, our findings demonstrated *E*. *granulosus* infection led to characteristics similar to those noted in cancer. Thus, the biological behaviors of *E*. *granulosus*, such as protoscolex dissemination and invasion in the intermediate host, development of protoscoleces into hydatid cysts, and finally, space-occupying lesion formation, may be similar to those involved in cancer cell proliferation and migration. These results may, therefore, aid in developing broad-spectrum blocking drugs.

In addition to *E*. *granulosus* invasion and migration (i.e., tumor-like biological behaviors), considerable changes were noted in various metabolic enzymes of the host, suggesting that *E*. *granulosus* cannot obtain nutrients and energy independently; this host dependence also provides potential candidate targets for drug development. The results demonstrated that *E*. *granulosus* affected the expression of host metabolic enzymes such as Hp, selenium-binding protein 2 (SBP2), pyruvate kinase (PYK) M (PKM), NADH-ubiquinone oxidoreductase chain 5 (MTND5).

Hp expression remained persistently and significantly downregulated in our model. Hp, synthesized by hepatocytes, participates in cellular oxidative stress response and is recognized and phagocytosed by macrophages after combining with free hemoglobin to form a stable complex [44,45]. This prevents hemoglobin accumulation from leading to kidney damage [46,47]. Clinically, in patients with liver cirrhosis, serum Hp expression can decrease; the degree of this decrease is closely related to disease severity [48]. In hepatocytes, Hp is a new ferroptosis inhibitor, which alleviates ERK1/2 phosphorylation [49]. Accumulating evidence suggests that Hp is an essential protein that maintains the liver’s normal physiological function. Our data demonstrated that the hepatocyte-secreted Hp levels decreased significantly, indicating that *E*. *granulosus* destroys the normal structure of the host hepatocytes and affects their related functions. The continuous changes in Hp can guide host liver lesion occurrence and development. As an acute phase protein (APP) [50], Hp may be a highly promising serum marker for predicting the severity of hidradenitis suppurativa, a chronic inflammatory disease [51]. As such, it may be a marker for early diagnosis of *E*. *granulosus* infection. In our infected group, the GO Biological Process category DEPs were enriched in amino acid metabolism and mainly occurred in the late infection stage. The liver is not only the main location for amino acid catabolism [52] but also the principal organ involved in hydatid production, including hydatid parasitization and growth. Moreover, severe liver injury and dysfunction in the late stage can directly lead to abnormalities in amino acid metabolism. As such, amino acid metabolism is associated with the progression of *E*. *granulosus* infection—consistent with the results of Zhu M *et al*. [53].

SBP2, another persistently downregulated DEP in our mouse model of *E*. *granulosus* infection, is mainly expressed in the liver. It specifically binds to selenium and acetaminophen [54] and regulates lipid metabolism through PPARA [55]. SBP2 downregulation has also been detected in mice with liver fibrosis [56] or nonalcoholic fatty liver disease [57]. Therefore, we believe that in a host, *E*. *granulosus* infection can lead to a blockade of lipid metabolism, possibly resulting in fatty liver and liver fibrosis development [58]. The involvement of SBP2 has been mentioned in the context of various liver diseases (including our animal models) [59–61]; however, the specific underlying mechanisms remain unknown. Nevertheless, SBP2 may be a promising target for lipid metabolic liver injury amelioration.

We detected PYK, another metabolism-related DEP, in our mouse model. PYK, a major regulatory enzyme involved in energy metabolism, mainly catalyzes irreversible steps in glycolysis, converting phosphoenolpyruvate to pyruvate and ATP [62,63]. In its microenvironment, a parasite strictly depends on the energy produced through glycolysis to maintain its own needs. In 1988, Roth *et al*. demonstrated that when the PYK content of *Plasmodium falciparum* was upregulated, the glucose consumption rate in the parasitic erythrocytes increased 100-fold [64]. This result indicated that glycolysis inhibition can effectively kill parasites. As an important regulatory enzyme in cellular glucose metabolism in parasite lifecycles, PYK has become a therapeutic target for infections of parasites including *P*. *falciparum*, *Trypanosoma*, *Leishmania*, and *Babesia microti* [65–68]. In our mouse model, we detected PKM, the expression of which increased persistently at all stages of infection. PKM is an isoenzyme of PYK, with M1 and M2 subtypes; PKM2 is typically expressed by cancer cells [69], and its upregulation can activate glycolysis and enhance malignancy [70]. Pharmacological targeting of PKM2 is considered a valuable approach for cancer therapy [71–73]; thus, screening of PKM2 inhibitors targeting metabolism regulation may aid in treating *E*. *granulosus* infection. Notably, a major abnormal metabolic signature called the Warburg effect [74] occurs in cancer cells; this effect impairs aerobic respiration, converting glucose and pyruvate into lactate, even with abundant oxygen. In this microenvironment, cancer cells demonstrated considerable proliferation, metastasis, and drug resistance [75–77]. PKM2 interacts with HIF1 in a hypoxic environment, promoting a cellular metabolic shift—from glucose oxidation to glycolysis—and playing a central role in metabolic abnormalities and inflammatory phenotypes [78]. We also identified a significantly reduced MTND5 abundance. In mitochondria, NADH-ubiquinone oxidoreductase complex I is a crucial electron transfer enzyme, which also plays a major role in cellular metabolism [79]: it can regulate reactive oxygen species production [80,81]. MTND5 can catalyze the synthesis of NADH-ubiquinone oxidoreductase complex I at an early stage [82], and *MTND5* deletion or mutation is associated with various mitochondrial diseases [83–86]. Persistent downregulation of MTND5 in our model suggested that *E*. *granulosus* infection can damage mitochondrial function in host cells, interfering with its redox and possibly leading to further changes in various physiological parameters or dysfunction of ion channels. Our proteomic data facilitated the identification of the pathways enriched with the several aforementioned metabolism-related proteins; as such, we detected differential (upregulated) expression (with gradually increasing FC) of PKM in lesions at all three stages of *E*. *granulosus* infection. In the late stage of the infection, the expression of the multiple proteins involved in oxidative phosphorylation decreased, whereas that of multiple key proteins, such as PFKM, ALDOA, and LDHB (all of which were selected for validation in this study), involved in the HIF1 pathway increased significantly. As such, similar to rapid tumor cell growth, rapid *E*. *granulosus* proliferation is associated with high energy demand, warranting increased uptake and consumption of glucose from the host. Moreover, the continuous accumulation of lactate and other intermediate metabolites, along with the possible metabolic reprogramming caused by pseudohypoxia, facilitates disease progression [87]. However, the underlying mechanisms warrant further exploration.

In conclusion, our mouse model demonstrated two major phenomena in the liver (the organ most commonly involved in *E*. *granulosus* infection): (i) Significant enrichment of proteins facilitating *E*. *granulosus* growth and development; these proteins may promote cyst formation and angiogenesis. (ii) Considerable inhibition of enzymes related to amino acid and lipid metabolism; moreover, abnormal host metabolism may result from parasite–host interactions such as nutrient competition or immune escape. These observations may facilitate the search for potential targets for early diagnosis and treatment of *E*. *granulosus* infection. However, in subsequent research, we will consider factors such as the effects of different disease models on *E*. *granulosus* infection diagnosis, as well as the potential adverse effects of treatment of targeted metabolic enzymes on normal host cells. We also noted that in the middle and late stages of the infection, oxidative phosphorylation was inhibited, and the expression of glycolysis-related proteins increased; thus, *E*. *granulosus* may promote metabolic transformation of the host. Therefore, we systematically analyzed the proteome in mouse liver lesions, indicating the mechanisms occurring within a host during CE progression and revealing possible hub regulators and pathways involved in parasite growth, development, and pathology with the host. Our data provide a rich resource for *E*. *granulosus* research, which can be mined using various approaches that are beyond the scope of the current study (see S4 Table for the entire data set). Our results also provide basic data for further in-depth exploration of key functional molecules involved in *E*. *granulosus*–host interactions.

## Supporting information

S1 Table Venn diagram of the DEPs. (XLSX)

S2 Table DEPs localized to the cytoskeleton and peroxisome. (XLSX)

S3 Table Partial proteomic data of validated proteins. (XLSX)

S4 Table All DEPs detected the three *E*. *granulosus* infection stages. (XLSX)

S1 Fig Observation of liver lesions in mice at different infection stages. Liver lesions were not evident in the early stage (1 month). White and translucent hydatid cysts appeared on the liver in the middle stage (3 months), whereas multiple protruding transparent cysts of different sizes were noted on the liver surface in the late stage (6 months).
(TIF)

S2 Fig Quality Control and Repeatability Testing of Samples. (A) Basic statistics of the MS data. Total spectra = the number of secondary spectra detected by a mass spectrometer. Matched spectra = the number of spectra matching the theoretical secondary spectrum. Peptides = the number of identified peptides. Unique peptides = the number of identified unique peptides. Identified proteins = the number of proteins resolved by unique peptides. Quantifiable proteins = the number of proteins changing in expression before and after *E*. *granulosus* infection, with their fold change calculated. (B) PCC for sample repeatability. (C) PCA was used to detect the repeatability of samples. (D) RSD for sample repeatability.
(TIF)

S3 Fig Subcellular Localization of DEPs in Liver Tissue from Mice Infected with *E*. *granulosus* at Different Infection Stages. (A) Upregulated DEPs 1 month after *E*. *granulosus* infection. (B) Downregulated DEPs at 1 month after *E*. *granulosus* infection. (C) All DEPs 1 month after *E*. *granulosus* infection. (D) All DEPs 3 months after *E*. *granulosus* infection. (E) All DEPs 6 months after *E*. *granulosus* infection.
(TIF)

S4 Fig GO and KEGG Pathway Enrichment Analysis of Identified *E*. *granulosus* Proteins. (A, B) GO enrichment analysis of downregulated DEPs at 3 (A) and 6 (B) months after *E*. *granulosus* infection in the Biological Process category. (C) DEPs enriched in multiple metabolism-related KEGG pathways significantly downregulated 6 months after *E*. *granulosus* infection. (D) Specific proteins (the expression of which increased significantly only 6 months after infection) in cluster 3 and their enriched KEGG pathways.
(TIF)

S5 Fig GSEA at Different *E*. *granulosus* Infection Stages. The *x*-axis represents the normalized enrichment score (NES), whereas the *y*-axis represents the specific pathways; red and green represent pathways enriched in upregulated (NES > 0) and downregulated (NES < 0) DEPs, respectively. Results at (A) 1, (B) 3, and (C) 6 months after infection.
(TIF)
